# Discrepancies between survey and administrative data on the use of mental health services in the general population: findings from a study conducted in Québec

**DOI:** 10.1186/1471-2458-11-837

**Published:** 2011-10-31

**Authors:** Aline Drapeau, Richard Boyer, Fatoumata Binta Diallo

**Affiliations:** 1 Département de psychiatrie de l'université de Montréal, C.P. 6138 Succ. Centre-Ville, Montréal, H3C 3J7, Canada; 2 Centre de recherche Fernand-Seguin, 7331 rue Hochelaga, Montréal, H1N 3V2, Canada; 3 Département de médecine sociale et préventive de l'université de Montréal, C.P. 6138 Succ. Centre-Ville, Montréal, H3C 3J7, Canada

## Abstract

**Background:**

Population surveys and health services registers are the main source of data for the management of public health. Yet, the validity of survey data on the use of mental health services has been questioned repeatedly due to the sensitive nature of mental illness and to the risk of recall bias. The main objectives of this study were to compare data on the use of mental health services from a large scale population survey and a national health services register and to identify the factors associated with the discrepancies observed between these two sources of data.

**Methods:**

This study was based on the individual linkage of data from the cycle 1.2 of the Canadian Community Health Survey (CCHS-1.2) and from the health services register of the Régie de l'assurance maladie du Québec (RAMQ). The RAMQ is the governmental agency managing the Quebec national health insurance program. The analyses mostly focused on the 637 Quebecer respondents who were recorded as users of mental health services in the RAMQ and who were self-reported users or non users of these services in the CCHS-1.2.

**Results:**

Roughly 75%, of those recorded as users of mental health services users in the RAMQ's register did not report using mental health services in the CCHS-1.2. The odds of disagreement between survey and administrative data were higher in seniors, individuals with a lower level of education, legal or de facto spouses and mothers of young children. They were lower in individuals with a psychiatric disorder and in frequent and more recent users of mental health services according to the RAMQ's register.

**Conclusions:**

These findings support the hypotheses that social desirability and recall bias are likely to affect the self-reported use of mental health services in a population survey. They stress the need to refine the investigation of mental health services in population surveys and to combine survey and administrative data, whenever possible, to obtain an optimal estimation of the population need for mental health care.

## Background

The combined use of population surveys and health services registers is a powerful tool for public health since their respective limitations and assets can balance each other. National health services registers are mostly implemented to manage the payment of health services. Therefore they can only supply data on to the population, services and health professionals covered by the health program. Population surveys provide data on topics that are not usually documented in health services registers and that can produce a more detailed description of services users. A potential limitation of survey data is their questionable validity when they involve sensitive issues.

The validity of survey data on the use of mental health services has been questioned repeatedly due to the sensitive nature of mental illness [1-4]. Indeed, prejudices against mental illness are widespread [5-7] and, as a consequence, people with a mental health problem are highly stigmatized [5-9]. Individuals with a negative attitude towards mental illness and those who have been victimized because of their mental illness or the mental illness of a close relative may be reluctant to report their symptoms and their use of mental health services in a population survey. However, although the prejudices against mental illness are widespread, they are not universal. They tend to be more prominent in some segments of the population and against specific psychiatric disorders. On the one hand, negative attitudes towards mental illness and towards people with mental health problems tend to increase with age [10-12] and to be more apparent for schizophrenia than for depression [10,11,13], in men than in women [10] and among singles and individuals living with a partner than in divorced, separated or widows [14]. On the other hand, they appear to decrease with education [12,14,15] and income [14,16] and to be uncommon in those exposed to mental illness as carers or because of a personal or family history of mental illness [10,12,17]. Findings regarding the influence of gender are conflicting; in some studies, women express more negative attitudes than men [11,15] whereas, in other studies, they appear more open-minded than men towards mental illness and toward the mentally ills [10,16,18]. Finally, no statistically significant association has been found between employment status and negative attitudes towards mental illness [12,16] despite the pervasive stigmatisation of people with mental health problems in the work environment [19,20].

In addition to the effect of social desirability, survey data on the use of health services may also be flawed by recall bias. In population surveys, the length of the time window used for the investigation of mental health services is traditionally the 12 months preceding the survey interview. Most individuals would find that recalling their use of health services in the past year is quite a challenge. Those with a declining memory, such as seniors [21] and individuals who have symptoms or who take medication that affect their memory [9,22] may be especially at risk of inaccurate recall. Bhandari and Wagner [21] carried out a meta-analysis of 42 studies based on the linkage of individual data from population surveys or patients-based studies and administrative registers. Only one of these focused on mental health services in the general population. Bhandari and Wagner [21] found that the probability of under-reporting health services was sizeable, that it increased with the length of the time window used to document these services (1 to 4 months: 26.0%; 6 months: 39.6%; 12 months: 50.3%) and that it was higher in seniors. They also noted that the reporting of health services was more accurate for salient services (e.g., hospitalisation vs. outpatient services) [21].

Although the comparison of survey and administrative data is the best available strategy to validate survey data on the use of mental health services, it is not perfect since it fails to recognise that consumers, services providers and decision-makers may not always agree on the definition of mental health services [1-3]. Traditionally, respondents are asked whether or not they have consulted a health professional in the past year for a mental health problem and, if they did, what category of health professional they have consulted. Thus, their answer depends, not only on their willingness or capacity to report their use of mental health services but also on their perception of their motive for their past consultation and on their definition of a mental health problem and of a mental health service. Services providers interpret the symptoms disclosed by their patients and classify them into one or more standardized diagnostics that may or may not fit with the patients' motives for the consultation. For example, a diabetic patient might consult a general practitioner (GP) on a regular basis. According to the patient, the motive for these consultations is diabetes. During a consultation, the patient mentions that he or she feels tired and nervous and does not sleep well. Upon further inquiry, the GP detects the first signs of depression but decide to not prescribe an anti-depressant for the moment. On his claim for payment, the GP records two diagnostics: diabetes and depression. The recording of a depression diagnostic is in line with the rules defined by decision-makers for the payment of health services since the patient's psychological symptoms were examined. Yet, the patient did not consult this health professional for a mental health problem and he or she was not treated by medication or therapy for a mental health problem. In all likelihood, this patient would not report this consultation as a mental health service in a population survey. This example is typical of occasional differences in the definition of mental health services by respondents and services providers that would generate a random variation in the discrepancy between survey and administrative data. In a public health perspective, the main issue is not so much random variation but rather systematic variations across specific segments of the population (e.g., those who are highly prejudiced against mental illness or who have a failing memory) since systematic variations raise some doubt regarding the validity of survey data on the use of mental health service.

The main objective of this study was to investigate the validity of survey data on the use of mental health services. More precisely, this study aimed to compare the use of mental health services recorded in a large scale population survey (cycle 1.2 of the Canadian Community Health Services Survey (CCHS-1.2)) and in a national health services register (register of the Régie de l'assurance maladie du Québec (RAMQ)). Given the hypothesised effect of social desirability and recall bias on the self-reported use of mental health services in surveys, systematic variations in the discrepancies between the CCHS-1.2 and the RAMQ were expected. Thus, a second objective was to identify the factors associated with the discrepancies vis-à-vis the use of mental health services between survey and administrative data. The selection of the factors under study was based, first, on the empirical evidence concerning factors associated with the prejudices against mental illness, the stigmatization of the mentally ills, and the under-reporting of mental health services in surveys compared to administrative registers and, second, on the assumption that a recent, salient or frequent use of mental health services was more likely to be recalled.

## Methods

### Sample

This study is based on the individual linkage of data from the CCHS-1.2 and from the RAMQ's health services register. The CCHS is a repeated cross-sectional population survey carried out by Statistics Canada. Cycle 1.2 of the CCHS was conducted in 2001-2002 and was designed to document the mental health of the population, the use of formal and informal mental health services and a number of factors traditionally associated with mental illness and with the use of mental health services. The target population was made up of individuals aged 15 years and more and living in private households. Respondents were randomly selected using a multistage stratified cluster sampling design. Additional information on the CCHS-1.2 can be found in Gravel et al. [23]. The RAMQ is the governmental agency managing the Quebec national health insurance program, which covers medical services provided by physicians (i.e., general practitioners and specialists) to resident Quebecers. The RAMQ's register is fed by the claims for payment of these physicians and the accuracy of RAMQ data is routinely ascertained to ensure that these claims are justified. The RAMQ register contains data on the patients (e.g., date of birth; sex; residence), the services (e.g., type of services; location; diagnosis) and the physicians (e.g., specialty) covered by the program.

**Figure 1:**
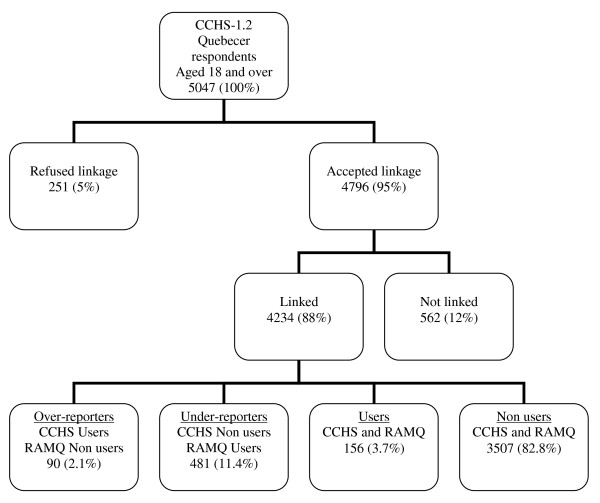
**Flow-chart of study participants**.

This study is restricted to Quebecer respondents aged 18 and more who agreed that the data that they had provided to the CCHS-1.2 be shared with governmental agencies for research purposes. The rate of agreement was high (n = 4796/5047: 95%) (Figure 1). Refusals were slightly higher in non employed (6.5%) than in employed (4.3%), in those without (5.6%) than with (4.1%) a spouse (legal or de facto) and in those with children in the household (6.0%) than without (4.4%) [24]. They did not differ by age, gender or the presence of a psychiatric disorder. Respondents aged 15 to 17 were excluded from this study because some of the questions related to the use of mental health services were not addressed to this segment of the population.

### Linkage procedure

Linkage of the data from the CCHS-1.2 and the RAMQ was carried out by the Institut de la statistique du Québec. It was based on the name, health insurance number, date of birth, sex of respondents and postal code of their residence. Most respondents (i.e., 4234/4796; 88%) were successfully linked with their RAMQ's record (Figure 1). Those who were not linked were more likely to be older than 45 years of age and to have a negative perception of their health. [24].

### Use of mental health services in the CCHS-1.2 and the RAMQ's register

In the CCHS-1.2, questions pertaining to the use of mental health services were addressed to all adult respondents whether or not they had experienced mental health problems in their lifetime or in the year preceding the survey interview. Respondents who reported talking to a general practitioner, a psychiatrist or another medical specialist for problems related to their emotions, their mental health or their drugs or alcohol intake in the twelve months preceding their interview in the CCHS-1.2 or following suicidal ideation or suicide attempt over the same period were coded as self-reported users of mental health services. Recorded use of mental health services in the RAMQ's register was based on three variables: medical specialty of the physician; type of medical act; and diagnosis. Individuals satisfying at least one of the following criteria in the twelve months preceding their interview in the CCHS-1.2 were coded as recorded users of mental health services: (1) health service provided by a psychiatrist; (2) psychiatric act (codes 360 to 394 of the RAMQ; these codes refers to psychiatric consultations, complete psychiatric examinations and psychiatric treatments in outpatient clinics or hospitals); (3) psychiatric diagnosis (codes 290 to 319 and 2900 to 3199 of the International Classification of Disease - 9th version (ICD-9)). The interval between each mental health service recorded in the RAMQ's register and the twelve month preceding the interview in the CCHS-1.2 was calculated individually for each respondent to ensure that the period of reference for the self-reported and recorded use was similar.

Self-reported use in the CCHS-1.2 and recorded use in the RAMQ's register were combined to produce four categories of respondents. Most respondents (82.8%) were non-users according to the CCHS-1.2 and the RAMQ's register; 2.1% were self-reported users but non users according to the RAMQ's register; 3.7% were self-reported and recorded users; and 11.4% were self-reported non users but recorded users (Figure 1). The analyses mostly focus on the last two categories (i.e. 637 respondents who were recorded as users of mental health services in the RAMQ's register and who were self-reported users or non users).

### Description of independent variables

Data on age, education (less than high school diploma; high school diploma; post-high school diploma; university diploma), employment status (with or without part-time of full-time employment), gender, marital status (with or without a legal or de facto spouse), parental status (with or without children aged 12 years or less in the household) were extracted from the CCHS-1.2 file.

Data on the psychiatric profile of respondents were provided by the CCHS-1.2 and the RAMQ's register. In the CCHS-1.2, psychiatric symptoms were elicited with the Composite International Diagnosis Interview (CIDI) for mood disorders (i.e., major depression and mania), and anxiety disorders (i.e., agoraphobia, panic attack, social phobia). The CIDI is based on the DSM-IV criteria. Three dichotomous variables were constructed from these data: presence/absence of self-reported symptoms compatible with a diagnosis of mood disorder; presence/absence of self-reported symptoms compatible with a diagnosis of anxiety disorder; neither mood nor anxiety disorder. In the RAMQ, diagnoses are specified by physicians on their claim for payment and are classified according to the ICD-9. Three dichotomous variables were created from these data: presence/absence of neurotic disorder; presence/absence of psychotic disorder; presence/absence of any other psychiatric diagnosis.

Recorded use of mental health services in the RAMQ's register was described by four variables: the number of mental health services provided in the year preceding the CCHS-1.2; the maximum number of criteria (i.e., service provided by a psychiatrist; psychiatric act; psychiatric diagnosis) satisfied in a single medical visit; the medical specialty of the physician (i.e., general practitioner; psychiatrist; other specialist); and the type of psychiatric act (i.e., consultation; complete exam; psychiatric treatment). In addition, the interval between the CCHS-1.2 interview and the most recent mental health recorded in the RAMQ's register was computed and split into four categories (1-3 months; 3-6 months; 6-9 months; 9-12 months).

### Statistical analysis

Percentages of agreement and disagreement between the CCHS-1.2 and the RAMQ data and Kappa inter-raters coefficients between these two sources of data were computed for the whole sample and for each variable under study. Logistic regressions were performed to estimate the odds ratio for disagreement vs. agreement between the CCHS-1.2 and the RAMQ's register for each variable. Confidence intervals were estimated at the 95% confidence level. All analyses were based on weighted data to control for non response and for the complex survey design of the CCHS-1.2. The weights were calculated by Statistics Canada.

### Ethical considerations

This study was restricted to respondents who agreed that the data that they had provided in the CCHS-1.2 be shared with governmental agencies for research purposes. Access to the CCHS-1.2 data file was granted by the Social Sciences and Humanities Research Council of Canada and Statistics Canada. Linkage of the CCHS-1.2 and the RAMQ files was authorized by the Commission d'accès à l'information du Québec. Data were analysed by the authors at the secured environment of the Centre d'accès des données de recherche of the Institut de la statistique du Québec (CADRISQ). This study was approved by the ethical committee of the Centre de recherche Fernand-Seguin of the Hôpital Louis-H. Lafontaine, which is the main research affiliation of the first author.

## Results

The sample was made up of 240 men and 398 women and the mean age was 49.3 (sd 15.4) years old. The mean number of recorded mental health services in the year preceding the survey was 4.1 (± 7.2) and 49.6% of respondents satisfied 2 or 3 criteria for a mental health service in a single medical visit. General practitioners were the most frequent mental health services providers (94.7%) and psychiatric treatment was the most frequent psychiatric act (71.0%). The interval between the CCHS-1.2 interview and the most recent mental health service recorded in the RAMQ's file was relatively short for 66.8% of respondents (1-3 months: 37.1%; 3-6 months: 29.7%). Additional descriptive data are presented in Tables 1 and 2.

**Table 1:** Socio-demographic and clinical description of the sample

	Agreement CCHS-RAMQ (n = 157)^a^	Disagreement CCHS-RAMQ (n = 481 )^a^	Total sample (n = 637)^a^
Socio-demographic profile			
Age			
Mean	42.85 ± 13.24	51.35 ± 15.53	49.26 ± 15.43
18-64 years	150 (95.5%)	370 (76.9%)	520 (81.5%)
65 and over	7 (4.5%)	111 (23.1%)	118 (18.5%)
Gender			
Men	50 (31.8%)	190 (39.5%)	240 (37.6%)
Women	107 (68.2%)	291 (60.5%)	398 (62.4%)
Education			
No high school diploma	30 (19.4%)	144 (31.0%)	174 (28.1%)
High school diploma	32 (20.6%)	93 (20.0%)	125 (20.2%)
Post-high school diploma	54 (34.8%)	142 (30.5%)	196 (31.6%)
University diploma	39 (25.2%)	86 (18.5%)	125 (20.2%)
Employment status			
Workers	103 (66.0%)	299 (62.3%)	402 (63.2%)
Non workers	53 (34.0)	181 (37.7%)	234 (36.8%)
Marital status			
With spouse	61 (39.1%)	295 (61.3%)	356 (55.9%)
Without spouse	95 (60.9%)	186 (38.7%)	281 (44.1%)
Parental status			
With young children	51 (32.7%)	132 (27.4%)	183 (28.7%)
Without young children	105 (67.3%)	349 (72.6%)	454 (71.3%)
Psychiatric disorder			
Based on CCHS-1.2 (DSM-IV)^b^			
Mood disorder	73 (46.5%)	27 (5.6%)	100 (15.7%)
Anxiety disorder	35 (22.2%)	19 (4.0%)	54 (8.5%)
Neither mood nor anxiety disorder	70 (44.6%)	439 (91.3%)	509 (79.9%)
Based on RAMQ (ICD-9)^a^			
Neurotic disorder	83 (52.9%)	190 (39.5%)	236 (37.0%)
Psychotic disorder	31 (19.7%)	16 (3.3%)	37 (5.8%)
Other psychiatric disorder	26 (16.6%)	55 (11.4%)	66 (10.4%)

a Un-weighted number of respondents. The other numbers in the Table are weighted.

b The percentages for psychiatric disorders are not additive since an individual can have more than one disorder

**Table 2:** Characteristics of the use of mental health services based on the RAMQ

	Agreement CCHS-RAMQ (n = 157 )^a^	Disagreement CCHS-RAMQ (n = 481 )^a^	Total sample (n = 637 )^a^
Number of mental health services			
Mean	8.61 ± 11.73	2.67 ± 3.92	4.12 ± 7.19
1	32 (20.4%)	215 (44.7%)	247 (38.7%)
2	21 (13.4%)	117 (24.3%)	138 (21.6%)
3 and more	104 (66.2%)	149 (31.0%)	253 (39.7%)
Number of criteria satisfied			
1	44 (28.2%)	277 (57.6%)	321 (50.4%)
2	75 (48.1%)	184 (38.3%)	259 (40.7%)
3	37 (23.7%)	20 (4.2%)	57 (8.9%)
Type of criterion satisfied^b^			
Specialty: psychiatry	40 (25.5%)	23 (4.8%)	63 (9.9%)
Psychiatric act	129 (82.2%)	352 (73.2%)	481 (75.5%)
Psychiatric diagnostic	139 (85.51%)	351 (73.0%)	490 (76.9%)
Medical specialty of physician^b^			
General practitioner	137 (87.3%)	466 (96.9%)	603 (94.7%)
Psychiatrist	40 (25.5%)	23 (4.8%)	63 (9.9%)
Type of psychiatric act^b^			
Consultation	23 (14.6%)	17 (3.5%)	40 (6.3%)
Complete exam	48 (30.8%)	36 (7.5%)	84 (13.2%)
Psychiatric treatment	119 (75.8%)	334 (69.4%)	453 (71.0%)
Interval between most recent mental health service and survey interview			
1-3 months	92 (59.4%)	144 (29.9%)	236 (37.1%)
3-6 months	39 (25.2%)	150 (31.2%)	189 (29.7%)
6-9 months	13 (8.4%)	100 (20.8%)	113 (17.8%)
9-12 months	11 (7.1%)	87 (18.1%)	98 (15.4%)

a Un-weighted number of respondents. The other numbers in the Table are weighted.

b The percentages for type of criteria, medical specialty of physician and type of psychiatric act are not additive since an individual can have satisfied different criteria, meet different physicians and had different act in different medical visits.

The one-year prevalence of the use of mental health services in adults was higher when estimated from the RAMQ's register (15%) than from the CCHS-1.2 (6%). The overall agreement between data from the CCHS-1.2 and the RAMQ was low (kappa = 0.29; CI_95% _0.25 to 0.34) for the use of mental health services and it was lowest in seniors (kappa = 0.05; CI_95% _0.00 to 0.12). In effect, 75.5% (481/637) of the respondents who, according to the RAMQ's register, had received a mental health service in the 12 months preceding their interview in the CCHS-1.2 report that they had not talked to a physician about their emotions, their mental health or their alcohol or drug problems during that period.

Several variables were associated with the discrepancy between the CCHS-1.2 and the RAMQ's register data (Table 3). For instance, the odds of disagreement decreased with education level and were higher in seniors and in respondents living with a partner. The lack of association with gender was unexpected so interactions between gender and the other socio-demographic variables were investigated. A significant interaction was found between gender and parental status: women and men who had no children at home did not differ in their odds of disagreement whereas a higher percentage of women (87%) than of men (63%) with young children did not report using mental health services although some mental health services were recorded in the RAMQ's register. The odds of disagreement were lower in respondents who self-reported symptoms compatible with a diagnosis of mood or anxiety disorder and in those who had a neurotic, psychotic or other psychiatric disorder according to the RAMQ's register.

**Table 3:** Odds ratio for disagreement vs. agreement between data from the CCHS-1.2 and the RAMQ's register

	Odds ratio	95% confidence interval
Age (ref., 18-64 years)	6.71	3.01 - 15.00
Gender (ref., Women)	1.40	0.956- 2.06
Education	0.78	0.66 - 0.93
Employment status (ref., Non employed)	0.85	0.58 - 1.24
Marital status (ref., Without a spouse)	2.48	1.72 - 3.60
Parental status (ref., (Without children in household)	0.77	0.52 - 1.14
Clinical profile (ref.: No psychiatric diagnosis)		
Based on CCHS-1.2		
Mood disorder	0.06	0.04 - 0.10
Anxiety disorder	0.09	0.05 - 0.16
Based on RAMQ		
Neurotic disorder	0.36	0.20 - 0.63
Psychotic disorder	0.11	0.05 - 0.22
Other psychiatric disorder	0.40	0.21 - 0.79
Number of recorded mental health services	0.44	0.35 - 0.55
Number of criteria satisfied in a single medical visit	0.32	0.24 - 0.42
Medical specialty of physician		
General practitioner (vs. other)	4.31	2.14 - 8.68
Psychiatrist (vs. other)	0.15	0.09 - 0.26
Type of psychiatric act		
Consultation (vs. other)	0.22	0.11 - 0.42
Complete exam (vs. other)	0.18	0.11 - 0.29
Psychiatric treatment (vs. other)	0.73	0.48 - 1.10
Interval between most recent mental health service and survey interview	1.90	1.55 - 2.33

As expected, discrepancies between self-reported and recorded use of mental health services were lower in individuals with a more frequent, more salient or more recent exposure to mental health services according to the RAMQ's register (Table 3). The odds of disagreement decreased with the number of recorded mental health services and with the number of criteria satisfied in a single medical visit and it increased with the length of the interval between the CCHS-1.2 interview and the most recent recorded mental health service. These odds were lower for services provided by a psychiatrist and higher for mental health services provided by general practitioners than for those provided by other physicians. They were also lower for psychiatric consultation and complete examination but were not statistically significant for psychiatric treatment.

Lastly, the over-reporting of mental health services was moderately high with 37% of self-reported users of mental health services having no recorded use in the RAMQ for the year preceding their interview in the CCHS-1.2. This over-reporting was high in seniors (OR = 4.5 CI_95% _1.7 to 12.1) but low in respondents with self-reported symptoms compatible with a mood disorder (OR = .32 CI_95% _0.15 to 0.70). Most cases of over-reporting were attributable to telescoping. Telescoping consists in reporting the use of mental health services that took place beyond the time window selected for the documentation of these services in the survey. Over-reporters were recorded as users of mental health services in the RAMQ in the 6 months (83.0% CI_95% _77.8 to 88.2) or 12 months (91.1% CI_95% _87.4 to 93.8) beyond the reference period of one year used in the CCHS-1.2.

## Discussion

Findings from this study show that the level of discrepancy between self-reported vs. recorded use of mental health services is high and that the odds of disagreement between the survey and administrative data under study are not random. The systematic variation of the discrepancies between survey and administrative data across specific segments of the population suggests that some biases, such as social desirability and recall bias, affect the validity of the self-reported use of mental health services in surveys. This study also shows that this disagreement is lower in individuals with a psychiatric disorder (either self-reported or according to the RAMQ) and in those recorded as frequent and more recent users of mental health services in the RAMQ register.

The limitations of this study must be kept in mind to better qualify these findings. First, as stressed previously, administrative data are not a perfect Gold Standard for the use of mental health services since patients may not be aware that a mental health service (as defined by services providers and decision-makers) has been provided. Second, this study is restricted to the mental health services and physicians covered by Quebec's national health insurance program. Thus findings from this study may not be generalized to services provided by other health professionals (e.g., psychologists and therapists) and to informal services (e.g., support groups). Among the 481 respondents who declared that they did not consult a physician for a mental health problem in the year preceding their interview in the CCHS-1.2, a small percent reported using other types of mental health service during that period (Psychologist: 5.0%; Informal service - e.g., Internet; help line -: 4.4%; Other health professional - Nurse or social worker -: 4.0%; Alternative medicine - e.g., Acupuncturist; massotherapist -: 0.4%). Third, in principle, the apparent under-reporting of mental health services in the CCHS-1.2 compared to the RAMQ's register could be partly attributable to the respondents' burden bias. Indeed to decrease the burden of respondents, it is customary to administer a series of questions on a specific topic only to respondents who answered positively to a filter question. When this strategy is used repeatedly, respondents learn to recognize the procedure and tend to answer negatively to a suspected filter question to avoid the burden of answering the ensuing series of question. In the CCHS-1.2, questions on the use of mental health services followed the questions on a number of topics (e.g., psychiatric symptoms for mood and anxiety disorders) that were introduced by a filter question using the same format (i.e., in your lifetime, have you ever...) as that used for the investigation of mental health services. Some respondents are likely to have recognised the filter question and to have answered negatively to avoid the load of questions on the use of mental health services. The influence of respondents' burden bias could not be investigated with the data at hand since the same questionnaire format was used for all respondents. However, the higher odds of disagreement between the CCHS-1.2 and the RAMQ in seniors, in less educated people, in individuals with a spouse and in women with young children go against the hypothesis of respondents' burden bias unless one assumes that these segments of the population are more likely to have recognized the filter question for the use of mental health services. Despite its limitations, this study has strengths that must also be taken into account in the appreciation of findings. In particular, the CCHS-1.2 is a population survey conducted by Statistics Canada instead of a study based on patients and the sample under study is sizeable. In addition, selection bias is unlikely to have affected the results since the refusal rate for the individual linkage of survey and administrative data for research purposes was low and the rate of successful linkage was high.

Some authors have hypothesised that individuals with a psychiatric disorder are more likely to conceal their use of mental health services in a survey interview for fear of the prejudices against mental illness or to inaccurately report their use of these services because they may take medication or have symptoms that affect their memory [3,9]. In contradiction with this hypothesis, a number of studies have shown that people with a personal experience of psychopathology are more open-minded about mental illness [10,12,14,17]. In this study, the odds of disagreement were indeed lower in respondents with a self-reported or recorded psychiatric disorder than in those without. In addition, the odds of disagreement between the CCHS-1.2 and the RAMQ's register was lower for psychotic disorder than for other psychiatric disorders although the empirical evidence show that the prejudices and stigmatisation tend to be higher for schizophrenia [10,11,13]. In agreement with the studies carried out among psychiatric patients by Heinrich et al, [25], Kashner et al. [26] and Killeen et al. [1], findings from this study suggest that the self-reported use of mental health services among individuals with a psychiatric disorder is largely consistent with the use recorded in administrative registers. The agreement between the CCHS-1.2 and the RAMQ's register was especially low among the respondents who had no mood or anxiety disorder according to their self-reported symptoms. In consequence, the accuracy of self-reported use of mental health services may be a public health concern not so much for individuals with a psychiatric disorder but rather for those who do not meet the diagnostic criteria (i.e., sub-clinical cases) or who do not report their symptoms for fear of the prejudice against mental illness.

In this study, the disagreement between survey and administrative data on the use of mental health services was higher for less educated individuals and people living with a partner. These findings tend to support the hypothesis that social desirability may affect the accuracy of self-reported use of mental health services since lower education [12,14,15] and living with a partner [14] have been associated with negative attitudes toward mental illness and the mentally ills. The higher odds of disagreement between the CCHS-1.2 and the RAMQ's register observed in women with young children than in men sharing the same family responsibility is somewhat puzzling. A tentative explanation may be that parenthood is more salient for women than for men so that the need for social approval regarding one's competency in the role of parent would be stronger for women than for men.

In agreement with the meta-analysis carried out by Bhandari and Wagner [21] of 42 studies based on the linkage of surveys data or patients reports and administrative data, the odds of disagreement between the CCHS-1.2 and the RAMQ were lower for more salient (i.e., services provided by psychiatrists), more frequent and more recent mental health services. Thus occasional and less recent services were less likely to be recalled. All but one of the studies reviewed by Bhandari and Wagner [21] focus on somatic illness thus suggesting that recall bias is a threat to the validity of survey data not only for the use of mental health services but also for the use of general health services. In this study, the hypothesis of recall bias is further supported by the higher odds of both under- and over-reporting in seniors, who are at higher risk of memory deficit, than in younger respondents. The higher odds of under-reporting in seniors is consistent with the findings of a study conducted by Rhodes and her colleagues [3,22], which was based on the linkage of data from a large scale population survey and data from the register maintained by the Ontario Health Insurance Program (OHIP). A number of strategies have been developed to foster the recall of past events in a research interview, including decreasing the length of the reference period and using a calendar showing major events that have occurred during that period. These strategies should be routinely applied in surveys dealing with the use of health services.

This study provides some support for the hypothesis that consumers and services providers or decision-makers may have a different perspective on the definition of mental health services. The point-of-views of patients, services providers and decision-makers regarding the nature (i.e., mental health vs. other health problem) of the health services received by patients are more likely to converge if these services are salient or frequent than if they are less significant or occasional. As expected, the disagreement between the CCHS-1.2 and the RAMQ was low for services provided by psychiatrists and it decreased with the number of services recorded as mental health services in the RAMQ's register. Patients who were not examined by a psychiatrist or who were recorded only one or twice as users of mental health services in a 1-year period may not have viewed these services as *mental health *services, especially if their motive for the consultation was not related to their mental health and if they were not treated (e.g., medication or therapy) for a mental health problem. Qualitative studies could contribute to clarify the consumers, providers and decision-makers point-of-views regarding the definition of a mental health service.

To our knowledge, this study and the study conducted by Rhodes and her colleagues [3,22] are the only published studies that have ascertained the accuracy of mental health services through the linkage of data from a population survey and a health services register. Both studies were conducted in Canada. Other studies comparing the self-reported use of mental health services and the use recorded in administrative registers were based on psychiatric patients [1,9,25,26] or Medicaid beneficiaries [27]. Our study replicates findings from these studies regarding the effect of age and psychiatric disorder on the accuracy of the self-reported use of mental health services. In addition, the profile of under-reporters observed in this study coincides with the profile of people with negative attitude towards mental illness in Canada and in other countries. Still, additional studies linking data from population surveys and administrative registers are needed to verify to what extent the under-reporting of the use of mental health services in population surveys is a widespread phenomenon.

## Conclusions

In conclusion, findings from this study stress the need to refine the investigation of mental health services in population surveys and to combine survey and administrative data, whenever possible, to obtain an optimal estimation of the population need for mental health care and a more detailed profile of the users of mental health services. Indeed, in all likelihood, the systematic variation observed in the discrepancies between the CCHS-1.2 and the RAMQ's register can, at least partly, be attributed to the pooled effect of social desirability and recall bias since the profile of under-reporters coincide with the general profile of people expressing negative attitudes towards mental illness and towards people with mental health problems (e.g., seniors; less educated individuals; people living with a partner) and of people with a memory deficit (e.g., seniors). Thus survey data would tend to under-estimate the use of mental health services in the population and to bias the profile of users. The next step would be to assess to what extent self-reported and recorded data differ in their estimation of the use of mental health services and in the description of the users.

## Competing interests

The authors declare that they have no competing interests.

## Authors' contributions

AD planned the study, reviewed the literature, supervised data analyses and the interpretation of findings, and assumed leadership for the writing of the manuscript. RB contributed to the planning of the study and of the analyses. FBD took part in the review of the literature and carried out the analyses. AD wrote the manuscript. RB and FBD participated in the interpretation of findings and commented the first version of the manuscript. All authors read and approved the final manuscript.

## Pre-publication history

The pre-publication history for this paper can be accessed here:

http://www.biomedcentral.com/1471-2458/11/837/prepub

## References

[B1] Killeen TK Brady KT Gold PB Tyson C Simpson KN Comparison of self-report versus agency records of service utilization in a community sample of individuals with alcohol use disorders Drug Alcohol Dependence 2004 73 141 147 10.1016/j.drugalcdep.2003.09.006 14725953

[B2] Marshall SF Deapen D Allen M Anton-Culver H Bernstein L Horn-Ross PL Peel D Pinder R Reinolds P Ross RK Validating California Teachers Study self-reports of recent hospitalization: Comparison with California hospital discharge data American Journal of Epidemiology 2003 158 1012 1020 10.1093/aje/kwg256 14607810

[B3] Rhodes AE Lin E Mustard CA Self-reported use of mental health services versus administrative records: should we care? International Journal of Methods in Psychiatric Research 2002 11 125 133 10.1002/mpr.130 12459825 PMC6878364

[B4] Taube CA Schlenger WE Rupp A Whitmore RW Validity of Medicaid household respondent reporting of ambulatory visits for mental disorders Journal of Economic and Social Measurement 1986 14 243 256 10302014

[B5] Kirby MJL Keon WJ De l'ombre à la lumière. La transformation des services concernant la santé mentale, la maladie mentale et la toxicomanie au Canada 2006 Canada: Comité sénatorial permanent des affaires sociales, des sciences et de la technologie

[B6] Hogan MF Achieving the Promise: Transforming Mental Health Care in America 2003 Maryland (USA): New Freedom Commission on Mental Health

[B7] Mental Health Commission of Canada Toward Recovery & Well-Being. A Framework for a Mental Health Strategy for Canada 2009 Ottawa (Ontario): Mental Health Commission of Canada

[B8] Corrigan PW Kleinlein P Corrigan PW The impact of mental illness stigma On the Stigma of Mental Illness 2005 Washington (DC): American Psychological Association 11 44

[B9] Hennessy KD Reed K Validating self-reports of mental health service use in a chronic population Journal of Nervous and Mental Disease 1992 180 399 400 10.1097/00005053-199206000-00011 1593276

[B10] Jorm A Griffiths K The public's stigmatizing attitudes towards people with mental disorders: how important are biomedical conceptualizations? Acta Psychiatr Scand 2008 118 315 321 10.1111/j.1600-0447.2008.01251.x 18759807

[B11] Lauber C Nordt C Falcato L Rossler W Factors influencing social distance toward people with mental illness Community Mental Health Journal 2004 40 3 265 274 15259631 10.1023/b:comh.0000026999.87728.2d

[B12] Van t' Veer J Kraan H Drosseart S Modde J Determinants that shape public attitudes towards the mentally ill: A Dutch public study Social Psychiatry and Psychiatric Epidemiology 2006 41 310 317 10.1007/s00127-005-0015-1 16501885

[B13] Link B Phelan J Bresnahan M Stueve A Pescosolido B Public conceptions of mental illness: Labels, causes, dangerousness, and social distance American Journal of Public Health 1999 89 9 1328 1333 10.2105/AJPH.89.9.1328 10474548 PMC1508784

[B14] Jagdeo A Cox BJ Stein MB Sareen J Negative attitudes toward help seeking for mental illness in 2 population-based surveys from the United States and Canada Can J Psychiatry 2009 54 11 757 766 19961664 10.1177/070674370905401106

[B15] Wang JL Fick G Adair C Lai D Gender specific correlates of stigma toward depression in a Canadien general population sample Journal of affective disorders 2007 103 91 97 10.1016/j.jad.2007.01.010 17292968

[B16] ten Have M de Graaf R Ormel J Vilagut G Kovess V Alonso J the ESEMeD/MHEDEA 2000 Investigators Are attitudes towards mental health help-seeking associated with service use? Results from the European Study of Epidemiology of mental Disorders Soc Psychiatry Psychiatr Epidemiol 2010 45 153 163 10.1007/s00127-009-0050-4 19381427 PMC2820660

[B17] Aromaa E Tolvanen A Tuulari J Wahlbeck K Attitudes towards people with mental disorders: the psychometric characteristics of a Finnish questionnaire Social Psychiatry and Psychiatric Epidemiology 2010 45 265 273 10.1007/s00127-009-0064-y 19436925

[B18] Gonzalez J Alegria M Prihoda T How do attitudes toward mental health treatment vary by age, gender, and ethnicity/race in young adults? Journal of Community Psychology 2005 33 611 629 10.1002/jcop.20071

[B19] Baldwin ML Marcus SC Perceived and Measured Stigma Among Workers With Serious Mental Illness Psychiatric Services 2006 57 3 388 392 10.1176/appi.ps.57.3.388 16524998

[B20] Baldwin ML Marcus SC Labor market outcomes of persons with mental disorders Industrial Relations 2007 46 481 510 10.1111/j.1468-232X.2007.00478.x

[B21] Bhandari A Wagner T Self-reported utilization of health care services: Improving measurement and accuracy Medical Care Research and Review 2006 63 217 235 10.1177/1077558705285298 16595412

[B22] Rhodes AE Fung K Self-reported use of mental health services versus administrative records: care to recall? International Journal of Methods in Psychiatric Research 2004 13 165 175 10.1002/mpr.172 15297900 PMC6878470

[B23] Gravel R Béland Y The Canadian Community Health Survey: Mental Health and Well-Being Canadian Journal of Psychiatry 2005 50 573 579 10.1177/070674370505001002 16276847

[B24] Baulne J Influence de l'ancrage social sur le comportement et l'attitude des femmes et des hommes par rapport à la maladie mentale: L'exemple de la sous-déclaration du recours aux services psychiatriques dans une enquête populationnelle. Projet d'appariement EPSEBE - Méthodologie et résultats Direction de la méthodologie et de la qualité 2009 Institut de la statistique du Québec

[B25] Heinrich S Deiser A Birker T Hierholzer C Weigelt I Zeichner D Angermeyer MC Roick C König H-H Accuracy of self-reports of mental health care utilization and calculated costs compared to hospital records Psychiatry Research 2011 185 261 8 10.1016/j.psychres.2010.04.053 20537717

[B26] Kashner TM Suppes T Rush AJ Altshuler KZ Measuring use of outpatient care among mentally ill individuals: a comparison of self reports and provider records Evaluation and Program Planning 1999 22 31 39 10.1016/S0149-7189(98)00038-X

[B27] Beebe TJ McRae JA Barnes SA A comparison of self-reported use of behavioral Health services with Medicaid agency records in Minnesota Psychiatric Services 2006 57 1652 1654 10.1176/appi.ps.57.11.1652 17085617

